# Usefulness of a Left Ventriculogram in Patients Presenting With Acute Coronary Syndrome Due to Takotsubo Cardiomyopathy: A Case Series

**DOI:** 10.7759/cureus.92298

**Published:** 2025-09-14

**Authors:** Zahid Khan, Konstantinos Tyrovolas, Paul Rees

**Affiliations:** 1 Cardiology, University of South Wales, Pontypridd, GBR; 2 Cardiology, University of Buckingham, London, GBR; 3 Cardiology, Barts Heart Centre, London, GBR; 4 Cardiology, Blizard Institute, Queen Mary University of London, London, GBR

**Keywords:** acute coronary syndrome, broken-heart syndrome, cardiogenic shock, cardiomopathy, left ventricular apical ballooning, left ventriculogram, myocardial infarction with no obstructive coronary atherosclerosis, postmenopausal complications, reverse takutsubo cardiomyopahty, takutsubo cardiomyopathy

## Abstract

Introduction: Takotsubo cardiomyopathy (TCM) is an acute and reversible cardiac condition in response to stress, characterised by apical ballooning of the left ventricle (LV) in the absence of coronary artery obstruction. Although the exact pathophysiology remains unclear, it is believed to be triggered by an endogenous surge of catecholamines in response to physiological or emotional stress. TCM patients generally experience a good recovery, and LV function typically recovers within two to three months in most patients.

Methodology: We present a case series of six patients who presented with chest pains and electrocardiographic (ECG) changes consistent with acute myocardial infarction (AMI). The selection criteria were patients presenting with chest pain, raised troponin I, and ECG changes suggestive of acute coronary syndrome (ACS).

Results: These patients ranged in age from their mid-50s to their mid-80s. All these patients presented with chest pain triggered by various activities, including watching a scary movie, adrenaline injection for anaphylaxis following consumption of pistachios, and the patient arguing with neighbours. All these patients underwent coronary angiograms that did not reveal any obvious culprit obstructive lesions, and a left ventriculogram (LVG) was performed in all six patients, demonstrating features of TCM. Most patients had cardiac magnetic resonance imaging (CMRI) as an outpatient, confirming the diagnosis of TCM. LVG showed classical apical ballooning in most patients presenting with TCM.

Conclusion: LVG can help diagnose TCM in patients with poor acoustic windows. This can be easily performed during coronary angioplasty in patients presenting with chest pain and suspected ACS.

## Introduction

Takotsubo cardiomyopathy (TCM) is characterised by left ventricular (LV) apical ballooning with transient systolic dysfunction in the absence of obstructive coronary artery disease (CAD) [1]. Patients mostly present with chest pain, shortness of breath, and electrocardiographic (ECG) changes consistent with acute coronary syndrome (ACS), and most patients tend to have elevated troponin I or T levels. These patients also have mild to moderate or occasionally severely impaired LV function, which usually recovers over time. Coronary angiography generally reveals unobstructed coronary arteries. Left ventricular outflow tract obstruction (LVOTO) is not common in patients with TCM; however, its early identification is crucial, as it can lead to cardiogenic shock, and traditional therapies can be detrimental in these patients [2,3]. It is essential to note that, unlike traditional cardiomyopathy, in which LV function may not recover, LV function in TCM tends to heal over time. It is more common in postmenopausal women, and the underlying pathophysiology is not entirely clear. However, a sudden surge in catecholamine release secondary to physical or emotional stress has been suggested to be responsible for this [4]. There are four recognised variants of TCM, with reported incidences of 81.7% (apical), 14.6% (midventricular), 2.2% (basal), and 1.5% (focal) [5]. Patients with apical-type have apical ballooning and basal hypercontraction; those with reverse TCM have basal akinesia and apical hypercontraction; those with mid-ventricular-type TCM have mid-ventricular ballooning and basal/apical hypercontraction; whereas localised or focal-type TCM can have any other segmental ballooning in the presence of LV dysfunction.

LV function recovers in most patients within a few months, and there is no difference in mortality based on sex or age. A minimal number of patients may develop serious and chronic complications, including recurrent TCM, persistent heart failure, cardiogenic shock, and death.

Backer et al. reported that the prevalence of LVOTO was approximately 20% in patients with troponin-positive TCM [3]. Mahmoud et al. reported the prevalence of TCM in their local population to be approximately 0.8%, based on data from 10,366 patients who underwent coronary angiography. Specifically, 32 patients were diagnosed with TCM, and 5% of patients did not have any significant coronary artery lesions [2]. Eight patients in this cohort of 32 developed LVOTO, resulting in a prevalence of 25%. Other studies have reported the prevalence of TCM to be approximately 1% to 2% in all patients presenting with ACS [6,7].

Left ventriculogram (LVG) is helpful in patients with TCM to reveal the pattern of regional wall motion abnormalities, such as apical or mid-ventricular segment (or both) akinesia of the left ventricle, as seen in classical TCM. Other patterns of LV wall motion abnormalities in patients with TCM include mid-ventricular akinesis with apical sparing, which has been described. This form is less common, as a recent report suggested a prevalence of approximately 40% [8,9]. LVG allows the assessment of nine LV segments to help differentiate TCM from ACS in most cases. Approximately 30% of patients with TCM and typical apical ballooning may have a very small zone of preserved LV contractility at the LV apex, known as the "apical nipple" sign [10]. This is not usually observed in patients with anterior acute myocardial infarction (AMI). Additionally, patients with mid-ventricular variant TCM can have a hawk’s beak appearance due to systolic contraction of the apex on ventriculography [10,11]. This case series presents six cases with poor acoustic windows on ECG and LVG, suggestive of TCM.

## Materials and methods

This is a retrospective case series of six patients presenting with AMI and LVG showing features of typical and atypical or reverse TCM conducted at Barts Heart Centre, London, United Kingdom. Data were collected from patients' electronic records, and images were obtained from the Picture Archiving and Communication System (PACS) at our hospital. These patients were admitted with signs and symptoms suggestive of AMI and underwent emergency coronary angiograms showing unobstructed coronary arteries. Data collection was conducted over six months from October 2024 to March 2025. Written consent was obtained from all six patients. 

## Results

Case 1

A 71-year-old woman presented to her local district general hospital (DGH) at 1700 hours with tongue swelling, shortness of breath, and tightness in her throat from 1200 hours after eating pistachios in a shop. Past medical history (PMH) was significant for hypertension (HTN), hypercholesterolaemia, and type 2 diabetes mellitus (T2DM). Regular medications included amlodipine 10 mg once daily (OD), reduced to 5 mg OD during admission, Humulin M3 (30:70) 100 units/mL, and Calcichew D3 Forte 400 units-1.25 gram oral chewable tablet, one tablet twice daily. She was given hydrocortisone 100 mg and chlorphenamine 10 mg IV. Her symptoms got worse, requiring 0.5 mg of adrenaline in a one in 1,000 (1 mg/mL) intramuscular solution in the emergency department three hours after presentation, which resulted in improvement in her symptoms and tongue swelling. Within minutes following the administration of adrenaline, patients developed central chest pain and tightness, and an ECG showed ST-segment elevation in the lateral leads and ST depression inferiorly. She was transferred to our cardiac centre for emergency coronary angiography and bedside echocardiography for mild left ventricular systolic dysfunction (LVSD) with apical hypokinesia. Coronary angiography via the radial artery showed unobstructed coronary arteries. The LVG at the end of the coronary angiogram demonstrated apical ballooning suggestive of TCM secondary to adrenaline administration for anaphylaxis (Video 1). Peak troponin was 3,529 ng/L (Table 1). Departmental ECG revealed a mild to moderate LVSD with an ejection fraction (EF) of 45%. She was started on bisoprolol 5 mg OD and ramipril 5 mg once daily, which was switched to candesartan 32 mg once daily due to a cough from ramipril, in addition to her regular medications. Cardiac magnetic resonance imaging (CMR) performed three months later showed normal biventricular function, and she was advised to continue her current medications (Video 2).

**Table 1:** Lab results of the patients on admission

Lab Test	Case 1	Case 2	Case 3	Case 4	Case 5	Case 6	Reference Range
Haemoglobin	12.2	12.6	12.2	9.0	129	134	13-17 g/dL
White cell count	21.6	9.8	4.6	12.4	9.3	8.9	4-10 x10^9/L
Neutrophil	19.9	6.1	2.6	11.4	6.7	7.0	2-7 x10^9/L
Platelet	234	212	195	323	226	203	150-410 x10^9/L
Sodium	135	142	142	137	138	138	133-146 mmol/L
Potassium	4.6	3.8	4.2	4.3	4.5	4.8	3.5-5.3 mmol/L
Urea	5.4	4.5	6.2	3.7	6.0	4.8	2.5-7.8 mmol/L
Creatinine	72	76	94	36	80	63	59-104 umol/L
Peaked troponin	441	3,529	2,149	297	161	370	0-14 ng/L
C-reactive protein	18	1	7	103	1	1	0-5 mg/L

**Video 1:** Left ventriculogram showing apical balllooning consistent with takotsubo cardiomyopathy

**Video 2:** Cardiac magnetic resonance imaging performed three months later shows recovered normal biventricular function.

Case 2 

A 63-year-old woman presented with the sudden onset of central chest pain following two days of nausea and vomiting due to food poisoning. PMH was significant for asthma only. Her regular medications include a beclomethasone inhaler twice daily. She was a lifelong smoker. The ECG showed anterior ST-segment elevation in leads V2-V6. The patient was administered aspirin 300 mg, clopidogrel 600 mg, morphine 5 mg IV, and 4 mg ondansetron IV. Emergency coronary angiogram showed unobstructed coronaries, and LVG showed moderately impaired LVSD with apical ballooning suggestive of TCM (Video 3). Peak troponin was 3,529 ng/L; other lab results are presented in Table 1. Echocardiography revealed moderately impaired LV systolic function, with an LVEF of 35%. The mid-septal and apical segments showed akinesia, and the mid-inferior and mid-anterior segments appeared hypokinetic (Video 4). The patient was commenced on dapagliflozin 10 mg OD, eplerenone 12.5 mg OD, ramipril 2.5 mg OD, and bisoprolol 3.75 mg OD. She did not tolerate CMRI due to claustrophobia and was discharged home after 48 hours of hospital admission. A repeat echocardiogram three months later showed an improvement in LV function to 45%. However, the patient was not compliant with medications and stopped taking them two months before her appointment. She ran out of medications during her overseas trip. Medication compliance was reiterated, and she remained compliant with medications following that.

**Video 3:** Left ventriculogram shows takotsubo cardiomyopathy.

**Video 4:** Echocardiography performed three months later shows improved left ventricular function, with an ejection fraction of about 45%.

Case 3 

An 83-year-old woman was transferred to our cardiac centre from a DGH after presenting with a sudden onset of chest pain associated with shortness of breath and palpitations in the evening. Chest pain was preceded by abdominal pain after attending to the toilet. PMH was significant for atrial fibrillation (AF), hypercholesterolaemia, and HTN. The regular medications included bisoprolol 1.25 mg OD, rivaroxaban (20 mg), atorvastatin (20 mg), and paracetamol. Before arrival at our centre, the subsequent ECG showed a posterior Q wave with some residual ST elevation. Bedside echocardiography revealed an EF of approximately 40% to 45%, with hypokinesia of the anterior and septal walls. Her initial troponin level was 2,149 ng/L. Emergency coronary angiography via right radial access using standard diagnostic 6Fr catheters revealed unobstructed coronary arteries, and LVG demonstrated apical ballooning with hypercontractility of basal segments, suggestive of TCM (Video 5). She commenced treatment with ramipril (2.5 mg) and spironolactone (12.5 mg), along with her regular medications. She was discharged from the ward after a few days of hospital stay and a peak troponin level of 2149 ng/L. CMRI imaging two months after discharge showed normal biventricular function and no evidence of scarring (Video 6). She was advised to continue her current medications and remained under cardiology outpatient follow-up.

**Video 5:** Left ventriculogram showing apical ballooning with hypercontractility of the basal segments

**Video 6:** Cardiac magnetic resonance imaging showing normal biventricular function

Case 4 

A 58-year-old Asian woman with multiple complex health conditions was admitted to our centre with suspected acute AMI following the sudden onset of chest pain radiating to her back while sitting on a couch at home. She reported a previous minor heart attack in 2009, but did not undergo percutaneous coronary intervention (PCI) at another centre. PMH was significant for HTN, T2DM, asthma, and epilepsy, paranoid schizophrenia, epilepsy, gastro-oesophageal reflux disease (GERD), obstructive sleep apnoea (OSA), and continuous positive airway pressure (CPAP). Regular medications included amlodipine 10 mg OD, hydrochlorothiazide 25 mg OD, moxonidine 100 mcg OD, bisoprolol 5 mg OD, hydralazine 25 mg OD, doxazosin 8 mg OD, spironolactone 25 mg OD, aspirin 75 mg OD, atorvastatin 20 mg OD, metformin 1 g twice daily (BD), and olanzapine 15 mg OD, although she was non-compliant with medications. The initial ECG showed an anterior ST-segment elevation myocardial infarction (STEMI); a bedside echocardiogram revealed apical hypokinesia with an estimated EF of 30%; departmental echocardiography also showed several LVSD cases with an EF of approximately 30%.

Emergency coronary angiography via right radial access using standard diagnostic catheters did not show any apparent coronary obstruction apart from moderate to severe distal disease in the first diagonal branch, and pre-dilatation was attempted with a 1 × 15 mm semi-compliant Trek balloon (Abbott Vascular, Abbott Park, IL, USA). Intravascular ultrasound imaging did not reveal any obvious left anterior descending (LAD) disease, and LVG showed severe LVSD and apical and anterior akinesia with apical ballooning, suggestive of TCM (Video 7). The peak troponin level was 1,284 ng/L. She became hypotensive the following day with reduced urine output, requiring admission to the intensive care unit (ICU) and raised inflammatory markers. She was commenced on 0.01 units/minute of vasopressin, milrinone 0.2 mcg/kg/min, IV co-amoxiclav 1.2 g three times a day (TDS), and clarithromycin 500 mg twice daily. The viral and atypical pneumonia screens were negative. Urine culture showed a white cell count of > 100 and mixed growth; the COVID-19 polymerase chain reaction (PCR) test was negative, and she was treated for possible urosepsis.

**Video 7:** Left ventriculogram showing features of takotusbo cardiomyopathy

She was discharged from the ICU two days later and was recommended regular medications. She could not undergo a cardiac MRI scan due to shrapnel in her lower abdomen from a previous accident. She was discharged home after two weeks of hospital stay on furosemide 20 mg OD, and the bisoprolol dose was reduced to 1.25 mg OD along with other regular medications. Echocardiography two months later showed a slightly improved LV function of 40%; her bisoprolol dose was increased to 2.5 mg OD, and spironolactone was increased to 50 mg OD (Video 8). The patient remains under the care of a cardiology outpatient clinic.

**Video 8:** Echo showing preserved biventricular function

Case 5 

A 55-year-old woman presented with the sudden onset of chest pain at about 1430 hours. She reported feeling sweaty, clammy, and nauseous. The chest pain was squeezing around the chest and lasted for over an hour. She presented to her local hospital, and the initial ECG showed a normal sinus rhythm; however, the repeat ECG revealed T-wave inversion (TWI) in leads V2-V6. Laboratory tests revealed a troponin T level of 161 ng/L. She was administered a loading dose of aspirin and clopidogrel and was transferred to our centre, given ongoing chest pain and a deep T wave in the anterolateral leads. PMH was significant for right breast cancer and had undergone mastectomy, chemotherapy, and radiotherapy, T2DM, and HTN. She had a smoking history of 20 pack-years and did not consume alcohol. Bedside echocardiography showed apical and septal hypokinesia with an LVEF of 45%. Regular medications included adjuvant abemaciclib, 1 mg anastrozole once daily, 10 mg amitriptyline once at night, metformin, 0.25 mg semaglutide once weekly, 40 mg omeprazole once daily, and 40 mg atorvastatin. Coronary angiography via right femoral access (RFA) revealed unobstructed coronary arteries, and the LV gram showed apical and septal hypokinesia, as well as apical ballooning (Video 9), consistent with TCM. She was advised to continue aspirin and clopidogrel and was booked for outpatient CMR that showed preserved biventricular function with no evidence of myocardial scarring (Video 10).

**Video 9:** Left ventriculogram showing features of takotsubo cardiomyopathy

**Video 10:** Cardiac magnetic resonance imaging showed preserved biventricular function

Case 6 

A 77-year-old lady presented to a DGH with a sudden onset of chest pain and palpitations while she went to speak to her neighbours to complain about the noise. She developed crushing chest pain that lasted for 30 minutes. PMH was significant for paroxysmal AF, HTN, and breast cancer, and she was awaiting the surgical removal of pre-cancerous cells. She had already undergone a lumpectomy and the removal of three lymph nodes a month prior. Usual medications included edoxaban 30 mg OD and bisoprolol 1.25 mg OD. The peak troponin level was 478 ng/L, and the ECG showed ST depression, T-wave changes, and borderline QT prolongation. She underwent an emergency coronary angiogram showing a mild atheroma in the left anterior descending, right coronary, and left circumflex arteries. The LVG showed an inferobasal aneurysmal segment suggestive of reverse TCM (Video 11). CMR showed a non-dilated LV, mildly impaired LV systolic function, and akinetic mid-to-apical inferior, mid-inferolateral, and mid-anterolateral segments without ischaemic scarring suggestive of reverse TCM. (Video 12). Echocardiography revealed low-normal LV function with an LVEF of 50%, accompanied by akinetic mid-inferolateral and mid-inferoseptal segments. Dual antiplatelet therapy was discontinued, and the patient was advised to continue edoxaban only. She was advised to continue ramipril and bisoprolol and was booked for outpatient CMR. The patient remained stable and was discharged with an outpatient follow-up. The laboratory test results are listed in Table 1.

**Video 11:** Left ventriculogram showing takotsubo cardiomyopathy

**Video 12:** Cardiac magnetic resonance imaging showing regional wall motion abnormalities and inferior wall oedema.

## Discussion

The true prevalence of TCM is unknown; however, it is estimated to range from 1% to 2% in patients presenting with troponin-positive chest pain or ACS [1,3]. It is more commonly seen in postmenopausal women, and its underlying aetiology seems to be a sudden adrenaline surge due to sudden emotional or physical stress. TCM often shows a pattern of regional wall motion abnormalities that encompasses areas beyond the perfusion territory of a single coronary artery. TCM has four different variants, and a study involving 62 patients with basal or inverted TCM showed a prevalence of approximately 2.2%. This form of the disease typically manifests in younger patients, with an average age of 36 years, compared to 62 years for other variants [12]. The International Takotsubo Diagnostic Criteria for TCM are similar to the Heart Failure Association (HFA) and European Society of Cardiology (ESC) criteria, except that the former excludes myocarditis. The HFA and ESC criteria for TCM include the absence of obstructive CAD that could explain the presentation, new and reversible ECG changes, elevated natriuretic peptide B-type (NT-proBNP) levels, elevated troponin levels, and recovery of LV systolic function within three to six months [5,12].

Certain ECG features are more sensitive for TCM than ACS, such as ST elevation in augmented vector right (aVR) combined with ST elevation in more than two to three anteroseptal leads (V1-V6), which is reported to have a positive predictive value of 100%, a negative predictive value of 52%, and a sensitivity of 12% [13]. Troponin T levels tend to be mildly elevated, whereas NT-proBNP levels tend to be much higher in patients with TCM than in those with ACS [5]. A serious complication of TCM is cardiogenic shock that may occur in the absence of LVOTO, and its management is primarily supportive. Occasionally, these patients may require mechanical circulatory support during the acute phase of cardiogenic shock; in the presence of LVOTO, this obstruction must be relieved before commencing heart failure treatment. Patients with TCM are at an increased risk of cardiac arrhythmias, and they should have at least 48 hours of inpatient cardiac monitoring.

Several case series and small-scale TCM studies have been published [1-11]. One study reported 11 patients with asthma exacerbation-induced TCM, and six of these 11 patients were postmenopausal women [5]. None of these patients had ECG findings that were more commonly associated with TCM than with ACS. Patients with classic TCM are more likely to present with pulmonary oedema, dyspnea, and cardiogenic shock than those with reverse TCM, which could be due to possible haemodynamic changes resulting from differences in the location of regional wall motion abnormalities. Similarly, LVOTO from left ventricular basal hypokinesia can contribute to mitral regurgitation or shock in patients with classic TCM [6,14]. The TCM registry study reported that the overall TCM-related mortality was approximately 4.1%, cardiogenic shock was 9.9%, and free wall rupture was approximately 0.5% [15-22]. Other possible complications include ventricular septal defect (VSD), mitral regurgitation, life-threatening arrhythmias, and LV thrombus [15]. The main risk factors for failure to warn (FWR) in both TCM and AMI include female sex, advanced age, presence of hypertension, and ST elevation, which is transient in TCM and resolves within a few days. Persistent ST elevation, very high levels of troponin and creatine kinase, elevated LV intramural pressure, and wall stress, as evidenced by an elevated LVOT gradient, are risk factors for FWR [15, 22-28].

Several case reports suggest that cyclin-dependent kinase (CDK) 4/6 inhibitors, either alone or in combination with letrozole and anastrozole, may predispose patients to TCM [29-32]. Chemotherapy-related cardiac toxicity is a rare but serious complication, and its exact frequency is unknown. Different chemotherapeutic agents have been reported to cause various degrees of myocardial dysfunction, and cyclin-dependent kinase (CDK) 4 and CDK6 inhibitors, such as abemaciclib, which are approved for the treatment of hormone receptor-positive breast cancer, have been reported to cause cardiomyopathy, including TCM [29,30].

Currently, there is a lack of standard management protocols for TCM patients. The International Takotsubo Registry study did not demonstrate a survival benefit from the use of beta-blockers; however, it reported improved one-year survival in patients treated with angiotensin-converting enzyme inhibitors (ACE-I) or angiotensin receptor blockers (ARBs) [28]. Our case series supports the findings of previous studies. Only one of our six patients developed cardiogenic shock, and most of our patients recovered completely.

Several imaging modalities can be used to diagnose TCM. The most commonly used modality is a transthoracic echocardiogram (TTE), which can help detect the distribution of regional wall motion abnormalities (RWMAs) and determine the morphological anatomical variant of TCM [10]. However, certain forms, such as the basal or focal types, can be challenging to diagnose with certainty on echocardiography alone. Strain imaging by speckle tracking echocardiography enables the assessment of global and regional myocardial function in all three layers of the myocardium, allowing for the evaluation of strain in the longitudinal, circumferential, and radial directions. It can also depict the transient impairment of myocardial deformation with circular involvement of the opposite LV walls (circumferential pattern). Speckle tracking should be performed in patients with TCM. Contrast echo can be helpful in patients with poor acoustic windows to determine the morphologic pattern of TTS. There is limited data to support the use of 3D echocardiography in patients with TCM. Both echocardiography and LVG have helped diagnose RWMAs in patients with TCM; however, CMR plays a vital role due to its ability to offer a comprehensive assessment of the functional and structural changes in patients with suspected TCM [10].

The OCTOPUS study, a prospective study involving 24 patients with TCM and 20 control patients, demonstrated a significantly increased left ventricular end diastolic volume (LVEDV) (P = 0.031) and left ventricular end systolic volume (LVESV) (P < 0.001) but a preserved LV stroke volume (P = 0.370) in patients with TCM [33]. Additionally, the LVEF was significantly reduced, and the heart rate was higher than in the TCM group compared to the control group. Similarly, patients with TCM were also more likely to have diastolic dysfunction with significantly elevated left ventricular end diastolic pressure (LVEDP) values in patients with TCM compared to the control group.

Limitations

This is a case series and lacks randomisation. Additionally, the sample size is very small; however, larger sample size studies are recommended. Additionally, the presentations in these patients varied depending on their exposure. 

## Conclusions

TCM is a reversible transient cardiomyopathy that is more common in postmenopausal women. Most patients tend to recover completely. However, some patients are at risk of serious complications, including cardiogenic shock. Most patients require careful monitoring for the first 48 hours during admission, and LV function typically recovers within three to six months in the majority of cases. The main risk factors for serious complications in TCM and AMI are similar and include female sex, advanced age, hypertension, and ST elevation.
